# Serpin Induced Antiviral Activity of Prostaglandin Synthetase-2 against HIV-1 Replication

**DOI:** 10.1371/journal.pone.0018589

**Published:** 2011-04-12

**Authors:** James B. Whitney, Mohammed Asmal, Ralf Geiben-Lynn

**Affiliations:** 1 Division of Viral Pathogenesis, Beth Israel Deaconess Medical Center, Boston, Massachusetts, United States of America; 2 Harvard Medical School, Boston, Massachusetts, United States of America; Academic Medical Center, Netherlands

## Abstract

The serine protease inhibitors (serpins) are anti-inflammatory proteins that have various functions. By screening a diverse panel of viruses, we demonstrate that the serpin antithrombin III (ATIII) has a broad-spectrum anti-viral activity for HIV-1, HCV and HSV. To investigate the mechanism of action in more detail we investigated the HIV-1 inhibition. Using gene-expression arrays we found that multiple host cell signal transduction pathways were activated by ATIII in HIV-1 infected cells but not in uninfected controls. Moreover, the signal pathways initiated by ATIII treatment, were more than 200-fold increased by the use of heparin-activated ATIII. The most up-regulated transcript in HIV-1 infected cells was prostaglandin synthetase-2 (PTGS2). Furthermore, we found that over-expression of PTGS2 reduced levels of HIV-1 replication in human PBMC. These findings suggest a central role for serpins in the host innate anti-viral response. Host factors such as PTGS2 elicited by ATIII treatment could be exploited in the development of novel anti-viral interventions.

## Introduction

Serine protease inhibitors (serpins) are elements of the innate immune system. They are part of the early physiologic response to viral infection that includes mannose binding lectins, soluble CD14, defensins, antimicrobial peptides, neutrophils, monocyte/macrophage and natural killer cells [1]. Serpins comprise a superfamily of genes that were originally found to regulate physiologic functions such as blood clotting, complement activation, programmed cell death, and inflammatory processes [2]. Biochemically, serpins belong to the largest and most diverse family of protease inhibitors [3]. Over 1000 serpins have been identified, including 36 human proteins, as well as molecules in plants, fungi, and bacteria [4], [5], [6].

Recently, links between serpins and human disease have been described. The anti-inflammatory activity of ATIII has been demonstrated in multiple anatomic tissues. In the lung, ATIII has been shown to inhibit neutrophil infiltration and decrease microvascular leakage [7]. In the liver, ATIII inhibits hepatic injury by regulating local prostacyclin levels [8], [9]. In the gastrointestinal tract ATIII attenuates leukocyte adhesion and rolling [10], and in the skin, ATIII reduces LPS-induced leukocyte-endothelial cell interaction [11]. Furthermore, ATIII has been found to influence the pathophysiology of atypical mycobacterial infection [12], diabetes mellitus [13], and panniculitis [14].

Serpins have been shown to play both positive and negative roles in the progression of chronic viral illnesses. Abnormally low alpha-1 anti-trypsin (AAT) levels in both HIV-1 [15], [16], and HCV infection have been shown to correlate with progressive disease or the development of liver fibrosis, respectively [17]. Conversely, there is mounting clinical evidence suggesting an association between increased levels of serpin expression and reduced incidence of HIV acquisition, or protracted disease progression [18], [19], [20].

In the present study, we demonstrated that heparin-activated ATIII elicit a potent anti-viral response by not only inhibiting HIV-1 as earlier described [20], [21] but also HCV, HSV-1 and HSV-2. We measured alterations in gene-expression pattern of HIV-infected PBMC after ATIII treatment to dissect the underlying mechanisms responsible for anti-viral activity of ATIII. We found that the PTGS2 protein was an integral component of an anti-inflammatory cascade. Our findings suggest a central role for PTGS2 in modulating the host innate response that could be exploited in the development of novel anti-HIV interventions.

## Materials and Methods

### Ethics statement

This study was reviewed and approved by the Human Research Ethics Committee of the Beth Israel Deaconess Medical Center (BIDMC) and Harvard Medical School (IRB 2006-P-000004). Written consent was waived since no personal data were collected.

### Source of ATIII

Plasma derived human ATIII (Talecris, Durham, NC) with a biological activity of 6 U/mg was used. ATIII protein was more than 98% pure, as determined by SDS-PAGE and silver staining, or by C4 high-pressure liquid chromatography (HPLC). ATIII was then activated by incubation with heparin as described (referred to as hep-ATIII). Briefly, ATIII was incubated with equal amounts (w/w) of heparin sodium (Polysciences, Warrinton, PA, cat. no. 01491) overnight at 37°C to form a non-covalent ATIII-heparin complex. Unbound heparin was then removed by gel-filtration with an TSK-gel G2000-SwxI column (Sigma-Aldrich, St. Louis, MO) as described [21]. Preparations resulted in less than 5% (w/w) free heparin.

### Large-scale screen of ATIII antiviral activity

The inhibitory hep-ATIII was determined using standard viral inhibition assays [22], [23], [24], [25], [26] against a panel of human pathogenic viruses and multiple subtypes that include: HIV-1, HCV, HSV-1, HSV-2, Measles, VEE, Tacarible Virus, SARS, Rift Valley Fever (MP-12), RSV A, Rhinovirus, PIV, New Guinea virus, Adenovirus (65089, Chicago), WNV and Dengue (New Guinea C) courtesy of the NIH biodefense program (http://www.niaid-aacf.org/screeningassays.htm). Inhibition of HCV replication was measured using the HCV OR6 replicon by the percentage of retained luciferase activity measured after ATIII treatment against an untreated control [27]. Luciferase activity was measured with the Renilla Luciferase Assay System (Promega, Madison, WI) for 1 sec. intervals using the 1420 Multilabel Counter Victor 3 (PerkinElmer, Waltham, MA).

Briefly, for each specific virus, drug concentrations in 5-fold increments were used in a virus-specific cell-based assay. From these data, the 50% inhibitory concentration (IC_50_), was calculated using the MacSynergy II Software [28]. Controls for inhibition experiments included vehicle buffer, bovine serum albumin (up to 30 µM) and a heparin control resembling the hep-ATIII purification step. Controls never reached more than 25% inhibition compared to untreated controls. A screen for drug toxicity was conducted in parallel. The 50% cytotoxic concentration (CC_50_) was determined on the basis of Neutral Red and Trypan blue (Sigma-Aldrich) stain exclusion. To determine if each compound has sufficient antiviral activity that exceeded its level of toxicity, a selectivity index (SI) was calculated according to CC_50_/IC_50_. An inhibition that had a SI of 10 or greater was considered to have useful anti-viral activity.

### Signal transduction pathway profiling

Hypaque-gradient purified human PBMC were stimulated for 3 days with 6.25 µg/ml concanavalin A (Con A) and then cultured in RPMI 1640 (Sigma-Aldrich) supplemented with 10% heat-inactivated fetal calf serum (FCS, Sigma-Aldrich), 10 mM HEPES, 2 mM glutamine, 100 U penicillin per ml, 10 mg of streptomycin per ml, and 50 U of IL-2 per ml (RPMI-50). PBMC (5×10^5^ cells/ml) were infected with a 0.01 multiplicity of infection (MOI) with primary isolate HIV 89.6 in a 96 well format [29] for 2 hours at 37°C. Afterwards, cells were washed twice and incubated with different amounts of ATIII or hep-ATIII in RPMI-50. Cells were harvested after 48 hr and total RNA was recovered using the RNeasy Kit (Qiagen) with a on-column DNAse digest (Qiagen) according to the manufacturer's protocol. Approximately 100 ng RNA was used for cDNA synthesis using the SuperArray RT^2^ First Strand Kit (SABiosciences, Frederick, MD, cat. no. C-03). cDNA synthesized was used for the RT^2^ Profiler PCR Array Human Signal Transduction PathwayFinder (SABiosciences, cat. no. PAHS-014A). The genes that were investigated can be found at http://www.sabiosciences.com/rt_pcr_product/HTML/PAHS-014A.html. Three arrays of three independent experiments were performed for each treatment condition. Relative levels of transcription were determined by using the ΔC_t_ values for each gene obtained by subtracting the mean threshold cycle (C_t_) of the GAPDH housekeeping gene from the C_t_ value of the gene of interest. Average ΔC_t_ value for 3 experiments was calculated, for each gene of interest, and average normalized transcription was calculated as follows: 2(-averageΔCt)^−1^. Fold increases of gene transcription, before and after treatment was calculated by dividing the average normalized transcription of each gene in the test samples by the corresponding control. Statistical significance in up- or down-regulation of transcription was determined by Student *t* test (2-sample, equal variance, 2-tailed distribution), comparing the ΔΔC_t_ (ΔΔC_t_ = ΔC_t_ treated - ΔΔC_t_ control). Significant differences were identified when *P* was less than 0.05.

### PBMC transfection and viral HIV-1 inhibition measurements

PBMC were hypaque-gradient purified and stimulated with 6.25 µg/mL ConA for 3 days. Cells were cultured in RPMI-50. PBMC (5×10^5^ cells/ml) were then transfected with the pCMV-PTGS2 construct (OriGene Technologies Inc.) using the PolyMag Magnetofection™method (OZBiosciences, Marseille, France) in RPMI-50 according to the manufacturer's protocol. Twenty-four hours after transfection PBMC were infected with a MOI of 0.01 with HIV 89.6 for 2 hrs, washed extensively and cultured at 37°C for 48 hrs in RPMI-50. To determine viral inhibition, HIV-1 RNA levels from cell culture supernatants were measured using the COBAS Ampliprep/COBAS Taqman 48 system (Roche, Indianapolis, IN) according to manufacturer's protocol and compared to controls.

### Analysis of protein interactive networks and statistical analysis

Functional analysis of interacting proteins was determined by using a commercial System Biology package, Ingenuity Pathways Analysis (IPA 8.0) (www.ingenuity.com) following the application directed protocols.

The statistical significance of differences between groups was determined using the program GraphPad Prism 4.0. A *P* value of <0.05 was considered statistically significant. Statistical analysis was performed by use of the Mann-Whitney test and the ΔΔC_t_ method.

### Western-blot analysis

Cells were homogenized by sonification. The protein concentration of the cell homogenate was determined with the Quick Start Bradford Dye Reagent (Bio-Rad, Hercules CA). Cellular homogenate was separated by SDS-PAGE and blotted as described [24]. Blots were developed as described [30] using a polyclonal PTGS2 antibody (Cell Signaling Technologies, Inc., Danvers, MA).

### PTGS2-ELISA

Peripheral blood mononuclear cells were isolated by Ficoll-Hypaque gradient centrifugation from whole blood of healthy human donors (Research Blood Components). PBMC were stimulated with 6.25 µg/mL Con A for 3 days. PBMC were then cultured in RPMI-50 and subsequently infected with HIV-1 89.6 at a multiplicity of infection of 0.01. Hep-ATIII was added to infected and uninfected control PBMC at concentrations of 0.09, 0.17 and 0.4 µM. After an additional 48 hours of incubation, cells were lysed using the CytoBuster Protein Extraction Reagent (Novagen/EMD Biosciences, Gibbstown, NJ), and PTGS2 was quantified using the PTGS2-specific ELISA Kit (EMD Biosciences, Gibbstown, NJ) according to manufacturer's instructions.

## Results

### Screen for ATIII-mediated inhibition of a diverse virus panel

Prior studies by our group have demonstrated the anti-HIV-1 activity of ATIII [20], [21]. To determine the potential range of ATIII-mediated antiviral activity we used a large-scale screen for inhibitory activity against a diverse panel of 15 human viruses. In this screen, we identified 3 pathogenic human viruses that were significantly inhibited by ATIII treatment, including HIV-1 (**Table 1**). The HIV-1 specific inhibitory activity of ATIII alone, while significant, required multiple-fold higher levels of ATIII than its normal 2.4 µM physiologic concentrations. However, the activation of ATIII with heparin (hep-ATIII) prior to its use in virus inhibition assays, dramatically increased its HIV-1-specific antiviral activity (**Table 1**). Unmodified ATIII had an IC_50_ for HIV-1 of 0.2–1 µM which was at least 3-fold higher than that of hep-ATIII. The difference in inhibitory activity between unmodified and modified ATIII was greater for several other viruses in the panel. For HCV, heparinization decreased the IC50 5.8-fold. Unmodified ATIII had no activity against HSV-1 and HSV-2, whereas hep-ATIII had IC50s of <6×10^−6^ and 5.3×10^−3^ µM respectively. Interestingly, the HSV-1 and HSV-2 IC50s were at least 10-fold lower than the ones for HIV and HCV. Importantly, hep-ATIII showed a clinically favorable selectivity index of up to >3000.

**Table 1:** IC_50_ of ATIII and hep-ATIII.

Serpin	Virus^a^	IC_50_ (µM)
ATIII	HIV^b^	0.2–1
	HCV^c^	43
	HSV-1	Not active
	HSV-2	Not active
Hep-ATIII	HIV	0.06
	HCV	7.3
	HSV-1	<6×10^−6^
	HSV-2	5.3×10^−3^

a Virus inhibition measured for the following viruses: HIV-1, HSV-1, HSV-2. No inhibition was observed for Measles VEE, Tacarible Virus, SARS, Rift Valley Fever (MP-12), RSV A, Rhinovirus, PIV, New Guinea virus, Adenovirus (65089, Chicago), WNV, Dengue (New Guinea C), and HCV.

b HIV-1IIIB with H9 T cell line was used.

c HCV replicon with full-length hepatitis C virus genome, strain O, was used.

### HIV-1 induced changes in cellular gene expression without drug treatment

**Figure 1:**
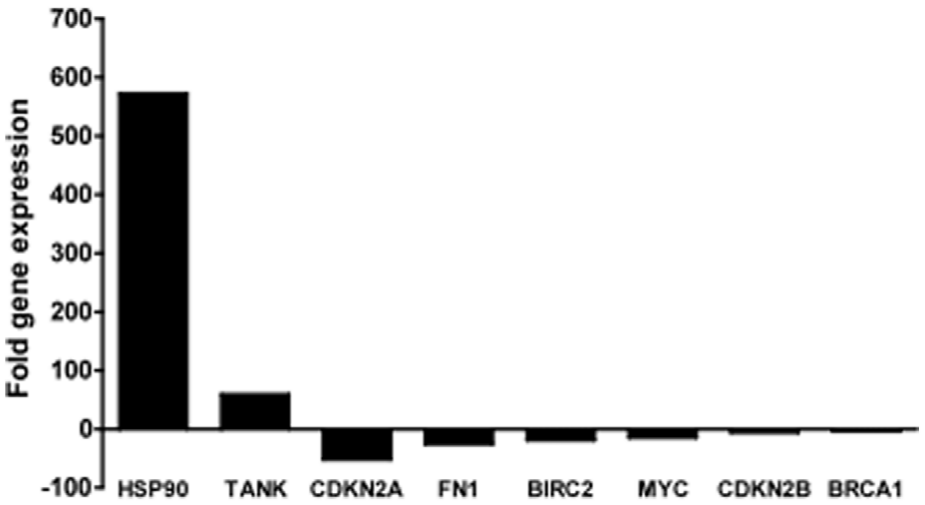
Effect of acute HIV infection on PBMC gene expression. Gene expression profiling of PBMC acutely infected with HIV-1. For signal transduction gene analysis, 10^5^ PBMC were infected with a 0.01 MOI of the primary isolate HIV-1 (HIV 89.6), for 2 h at 37°C. Total RNA was purified two days after infection. A RT-PCR expression array was performed and gene expression of 84 genes from 20 different signal transduction pathways was analyzed. Genes with significant changes in gene expression (*p*<0.05, n = 3) compared to uninfected vehicle treated controls are shown. Significance was calculated using the ΔΔC_t_ method for three independent experiments.

We sought to elucidate ATIII's mechanism of action underlying ATIII viral inhibition using gene expression profiling of well-characterized cellular signal transduction pathways. We screened for alterations in gene expression as a result of ATIII or hep-ATIII treatment in 20 different signal transduction pathways (totaling 84 independent genes) using a RT-PCR-based gene array. We first investigated the effect of HIV-1 infection alone to establish a baseline of transcriptional profiles that were associated with early viral replication. Compared to vehicle treated PBMC, we found that within 48 hrs of infection, HIV significantly altered the regulation of 8 genes from 5 signal pathways (**Fig. 1**). The heat-shock protein pathway was prominently altered, with heat-shock protein 90 (HSP90) expression increased to more than 500-fold over basal levels (*P* = 0.02). Interestingly, the expression levels of 2 genes from the NF-κB pathway were also altered; TRAF family member-associated NFκB activator (TANK) was increased 50-fold, whereas the expression of the anti-apoptotic baculo-viral IAP repeat-containing-2 gene (BIRC2) diminished approximately 10-fold (*P* = 0.04). We also found that genes fibronectin 1 (FN1) (*P* = 0.03) and myelocytomatoxis viral oncogene homolog (Myc) (*P* = 0.03) of the phosphoinositide-3 (PI3) kinase/AKT pathway were down regulated 10-fold (**Fig. 1**). Moreover, expression levels of the two cyclin-dependent kinase inhibitors, CDKN2A and CDKN2B, of the transforming growth factor- (TGF) pathway were up to 10-fold decreased with *P* of 0.02 and 0.03, respectively. Finally, we found an up to 5-fold (*P* = 0.002) down-regulation of the expression of the tumor suppressor and DNA repair gene breast cancer type 1 susceptibility protein (BRCA1) (**Fig. 1**).

### ATIII-induced gene expression changes in uninfected PBMC

**Figure 2:**
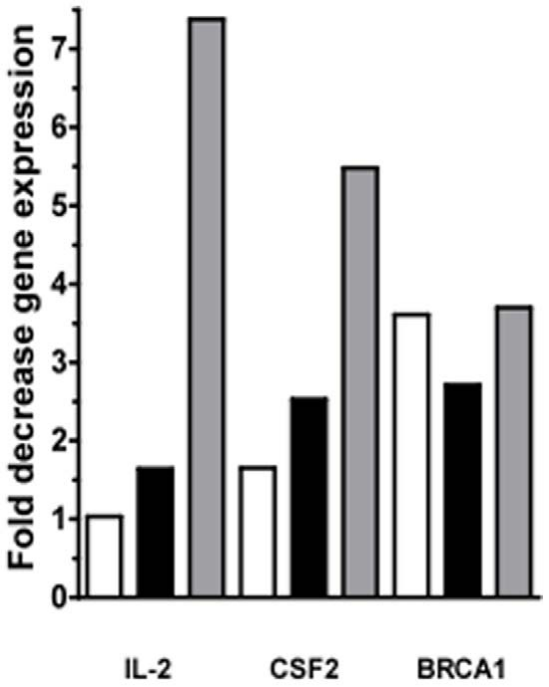
Effect of ATIII on gene expression in uninfected PBMC. Gene-regulation of uninfected PBMC treated with different doses of ATIII, each dose compared to an uninfected, ATIII untreated vehicle control. For signal transduction gene analysis, 10^5^ PBMC were infected with a 0.01 MOI of primary isolate HIV-1 (HIV 89.6) for 2 h at 37°C. Cells were washed and treated with 6.8, 34 and 68 µM ATIII for 48 h. Total RNA was purified and a RT-PCR expression array was performed. The expression of 84 genes from 20 different signal transduction pathways was analyzed. Genes with significant changes in gene expression (*p*<0.05, n = 3) compared to controls are shown. Significance was calculated using the ΔΔC_t_ method for three independent experiments.

We also tested the effect of ATIII alone on cellular gene expression in human PBMC to determine transcriptional changes affected by ATIII in the absence of HIV infection. We found that compared to vehicle treated controls, the levels of transcription of both interleukin 2 (IL-2) and colony stimulating factor-2 (CSF2), were altered in a dose-dependent manner up to more than 7- (*P* = 0.02) and 5-fold (*P* = 0.0002), respectively. Additionally, BRCA1 expression was more than 3-fold (*P* = 0.01) decreased (**Fig. 2**).

### ATIII-induced alterations in gene expression in HIV-1 infected PBMC

**Figure 3:**
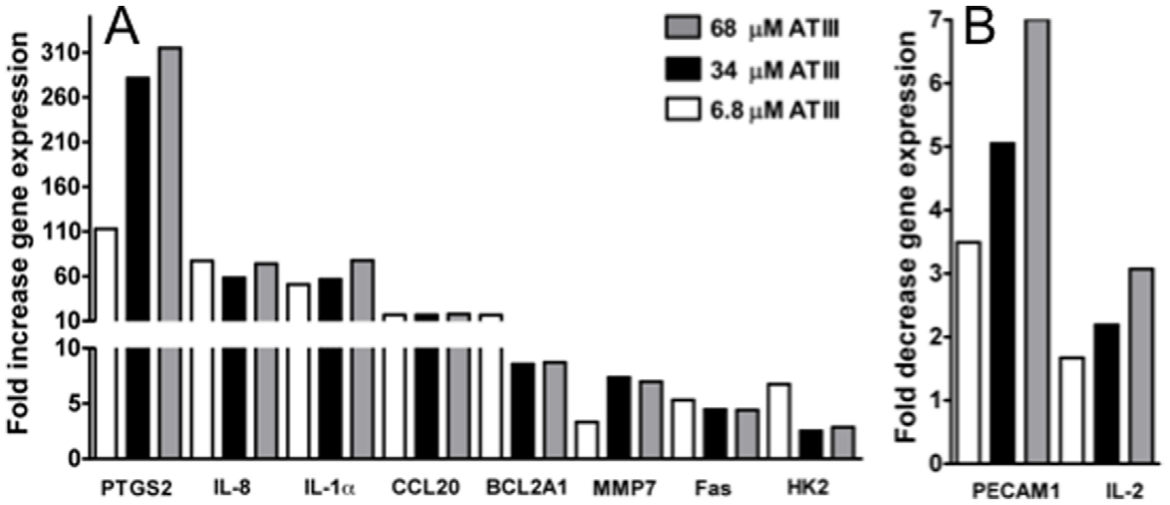
Effect of ATIII on gene expression in acutely HIV infected PBMC. (**A**) Gene up-regulation of acutely infected PBMC after treatment with different doses of ATIII, each dose compared to infected untreated control. (**B**) Gene down-regulation of acutely infected PBMC after treatment with different doses of ATIII, each dose compared to an infected untreated control. For signal transduction gene analysis, 10^5^ PBMC were infected with a 0.01 MOI of primary isolate HIV-1 (HIV 89.6) for 2 h at 37°C. Cells were washed and treated with 6.8, 34 and 68 µM ATIII for 48 h. Total RNA was purified and a RT-PCR expression array was performed. The gene expression levels from 20 different signal transduction pathways were analyzed. Genes with significant changes in gene expression (*p*<0.05, n = 3) compared to controls are shown. Significance was calculated using the ΔΔC_t_ method for three independent experiments.

We evaluated the effect of 6.8, 34 and 68 µM ATIII on gene expression in selected signal transduction pathways in HIV-1-infected PBMC. We identified several genes whose expression was affected by ATIII treatment during HIV infection when compared to the vehicle treated controls, but which were not perturbed by HIV-1 replication in the absence of ATIII (**Fig. 1**) or by ATIII treatment of uninfected cells (**Fig. 2**). We found that when compared to the untreated control the transcription of IL-8 and IL-1α were increased both more than 75-fold (*P* = 0.0008 and *P* = 0.0009, respectively); chemokine C-C motif ligand 20 (CCL20) was more than 17-fold increased (*P* = 0.02). In contrast, platelet/endothelial cell adhesion molecule (PECAM1, CD31 antigen) was 7-fold (*P* = 0.002) and IL-2 was more than 3-fold down-regulated (*P* = 0.049) at the highest ATIII concentration used (**Fig. 3A and B**).

Interestingly, PTGS2 was the most dramatically up-regulated of all genes investigated. When ATIII was added at 2.5-fold its physiological concentration, 6.8 µM, PTGS2 gene expression levels increased dramatically to 110-fold (*P* = 0.0001) over baseline levels. At the highest concentration of ATIII tested, PTGS2 transcription was increased to over 315-fold (*P*<0.0001) of control expression levels (**Fig. 3A**). Other signal transduction pathways affected included the p53 pathway with the Fas receptor, up to 5-fold increased (*P* = 0.02), and the PI3 kinase/AKT pathway with matrix metalloproteinase 7 (MMP7) and hexokinase 2 (HK2), both up to over 5-fold up-regulated (*P* = 0.001 and 0.047, respectively) (**Fig. 3A**).

### Heparin activated ATIII-induced host genes in HIV-1 infected PBMC

Non-activated ATIII is only a weak inhibitor of HIV-1 replication on its own. Multiple magnitudes of its 2.4 µM physiological concentrations are needed to achieve high viral (>90%) inhibition. Heparin activation of ATIII potently increases its anti-viral activity. Therefore, we investigated if this activation also has an effect on genes altered by ATIII during acute infection. We hypothesized that since anti-viral activity is increased by hep-ATIII, genes responsible for its viral inhibition might also be increased. Based on our data of the relative viral inhibitory capacities of ATIII versus hep-ATIII, we determined that we could use 200-fold less hep-ATIII (by concentration) as compared to ATIII alone to elicit equivalent changes in gene transcription and subsequent viral inhibition.

**Figure 4:**
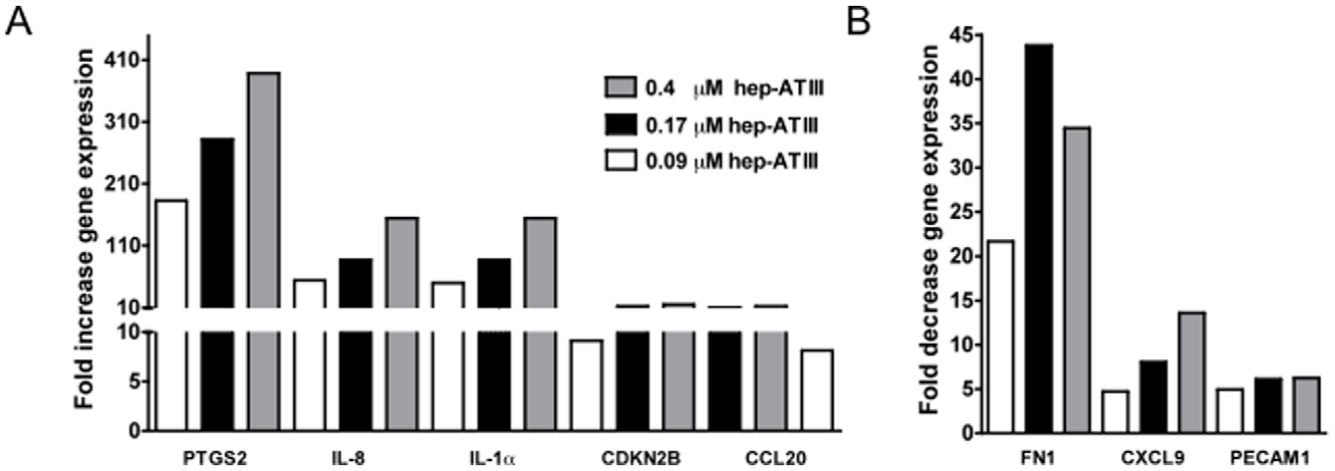
Effect of heparin activated ATIII (hep-ATIII) on gene expression in acutely HIV infected PBMC. (**A**) Gene up-regulation of acutely infected PBMC after treatment with different doses of hep-ATIII, each dose compared to infected hep-ATIII untreated vehicle control. (**B**) Gene down-regulation of acutely infected PBMC after treatment with different doses of hep-ATIII, each dose compared to infected hep-ATIII untreated vehicle control. For signal transduction gene analysis, 10^5^ PBMC were infected with a 0.01 MOI of primary isolate HIV-1 (HIV 89.6) for 2 hrs at 37°C. Cells were washed and treated with 0.09, 0.17 and 0.4 µM hep-ATIII for 48 h. Total RNA was purified and a RT-PCR expression array was performed. The expression levels of genes of 20 different signal transduction pathways (84 genes) were analyzed. Genes with significant changes in gene expression (*p*<0.05, n = 3) compared to controls are show. Significance was calculated using the ΔΔC_t_ method for three independent experiments.

Using 0.4 µM of hep-ATIII, we found the same genes up-regulated PTGS2 (P = 0.0007), IL-8 (P<0.0001), IL-1α (P<0.0001) and CCL20 (P<0.0001), or down-regulated PECAM1 (P = 0.0013) (**Fig. 4A and 4B**) as with a much higher dose of non-activated ATIII treatment. Overall, this confirmed our hypothesis that the enhanced viral inhibition mediated by hep-ATIII is largely achieved by similar transcriptional regulation as was effected by non-activated ATIII. Again, hep-ATIII activated PTGS2 potently, more than 380-fold at 0.4 µM when compared to untreated infected controls.

Interestingly, several genes did exhibit different transcriptional changes with hep-ATIII treatment compared to non-activated ATIII. Specifically, CDKN2B gene activity (*P* = 0.0005 *at 0.4* µM) was increased (**Fig. 4A**), whereas FN1 (*P* = 0.005 *at 0.4* µM) and chemokine (C-X-C motif) ligand 9 (CXCL9) (*P* = 0.0001 *at 0.4* µM) were transcriptionally down-regulated (**Fig. 4B**). We attribute these differences to the increased biological activity of the heparin-activated ATIII derivative.

### Network analysis of ATIII-induced interactomes during HIV-1 replication

**Figure 5:**
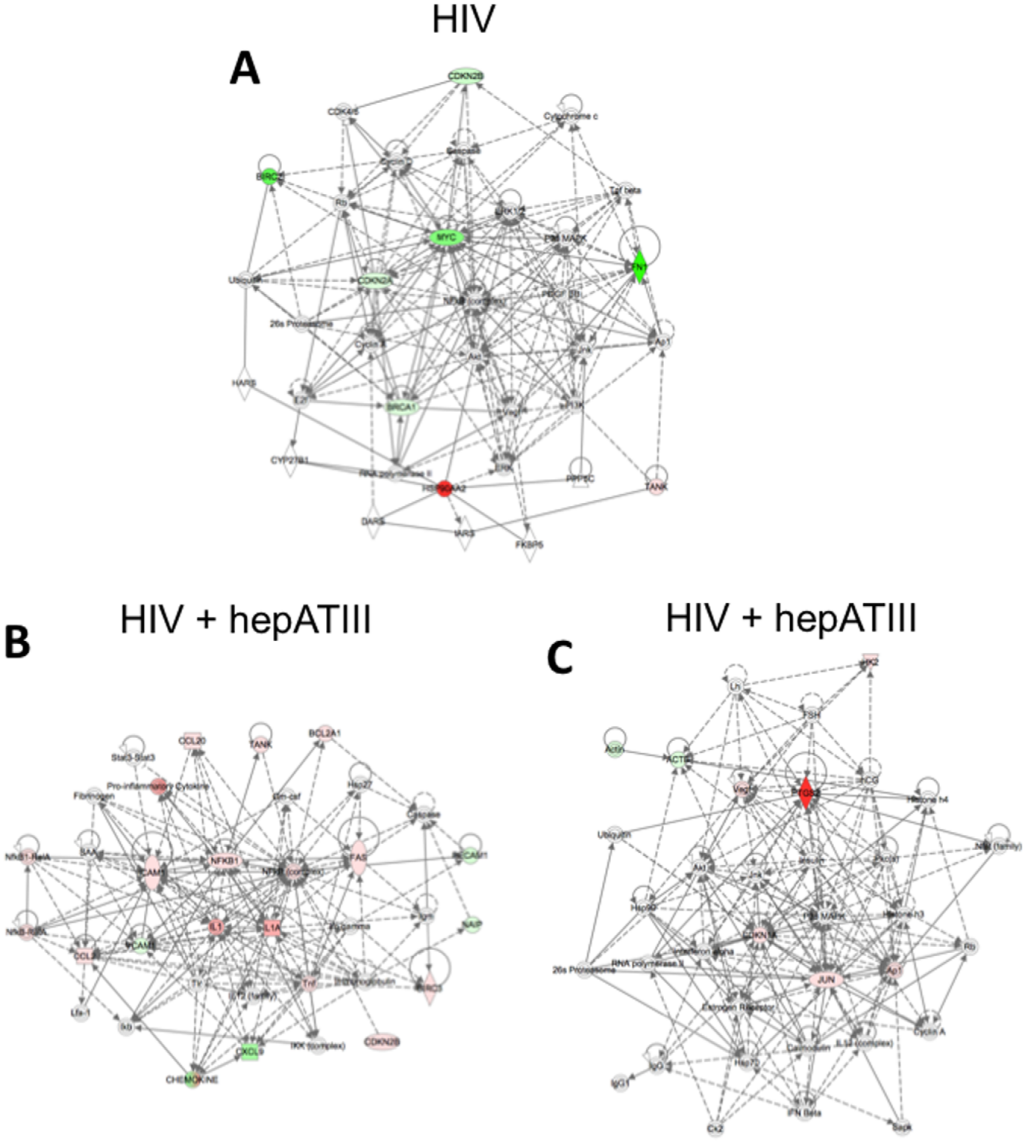
Interactive network analysis in acutely HIV-1 infected PBMC. (**A**) Effect of HIV-infection on gene expression in PBMC without hep-ATIII treatment. (**B**) The highest scored primary network activated by hep-ATIII in HIV-1 infected PBMC. (**C**) The secondary overlapping network activated by hep-ATIII in HIV-1 infected PBMC. Pathway analysis of significant up-regulated (red and down-regulated (green) genes in HIV-1 infected PBMC using Ingenuity Pathways Analysis Software. Significance was calculated using the ΔΔC_t_ method for three independent experiments.

To gain further insight into the mechanism of action of hep-ATIII in reducing HIV replication, we performed a biologic network analysis. This analysis method complements data generated from our gene arrays by facilitating the recognition hierarchical gene clusters that intersect with HIV replication. Using this method, we analyzed the effect of HIV-1 infection on PBMC in the absence of ATIII treatment. We found the NF-κB node of proteins scored significantly during HIV infection, underscoring the well-described role of these proteins in HIV RNA transcription. We found that 6 genes were significantly altered, and all intersect the NF-κB node. Three of these genes baculoviral IAP repeat containing 2 (Birc2), Myc and FN1 are strongly down-regulated, and CDKN2A, CDKN2B and BRAC1 are moderately down-regulated during acute HIV-1 infection (**Fig. 5A**). The highest increase in transcription was observed with HSP90-AA2, connected with the extracellular signal-regulated kinases (ERK) and ERK1/2 node during HIV infection.

We then examined the effect of hep-ATIII treatment in HIV infected cells. Using the same analysis method, we found a network with several genes and gene clusters that changed more than 2-fold during 48 h of hep-ATIII treatment. We found that most genes are connected with the NF-κB nodule (Fig. 5B): Highly activated was the cytokine IL-1, moderately altered are ICAM1, CDKN2B, TNF, BIRC3, Fas, Tank, BCL2A1, chemokine (C-C motif) ligand 2 (CCL2) and CCL20. There were also genes down-regulated dependent on the NF-κB nodule: CXCL9 and vascular cell adhesion molecule 1 (VCAM1) were significantly down-regulated (**Fig. 5B**). Other nodules in the same network were IL1, Tnf and the Nf-κB1-RefA dimer.

There is also another network activated by hep-ATIII treatment during HIV infection (**Fig. 5C**) which includes proteins dependent on the activator protein (Ap1) transcription factor, including PTGS2 (highly up-regulated), CDKN1A, and Jun. Intersecting this network is Vegf which regulates PTGS2. Moreover, several of these proteins intersect with the insulin node that regulates the anti-inflammatory immune responses of other serpins [31].

### Over-expression of PTGS2 affects HIV-1 replication

**Figure 6:**
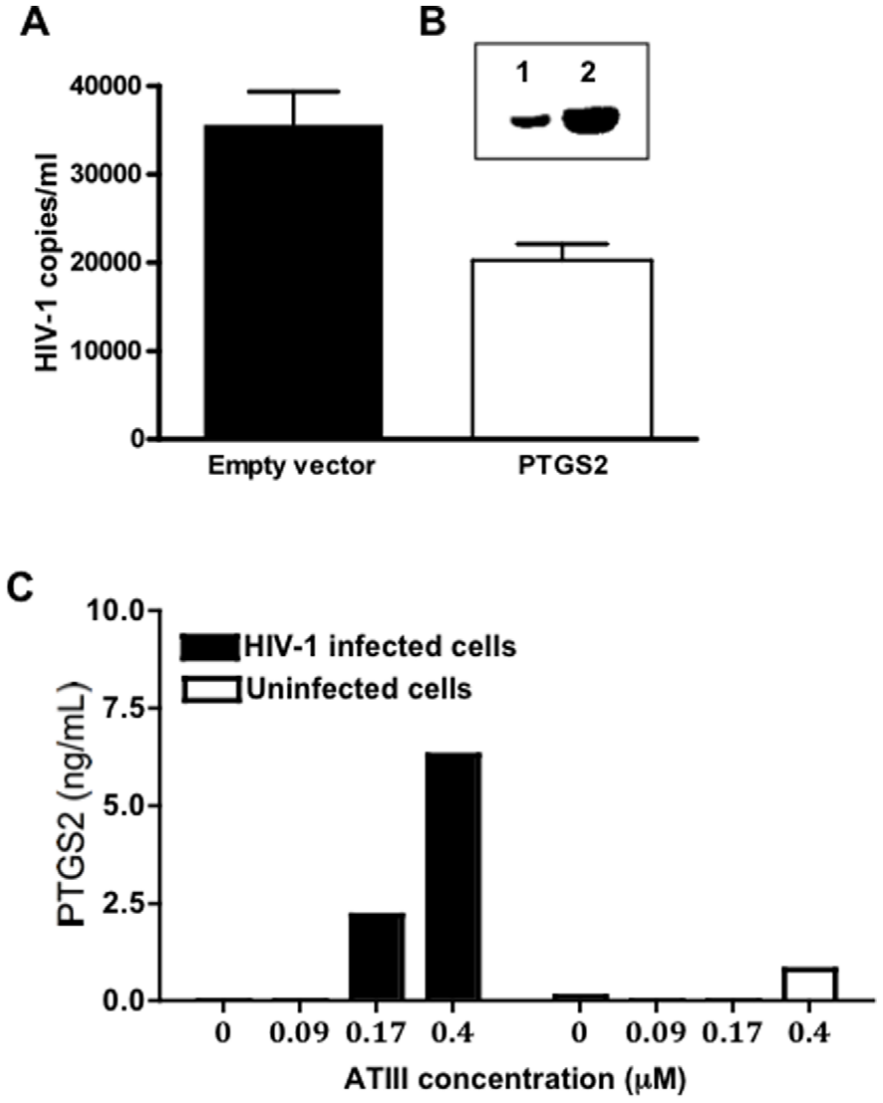
Inhibition of HIV replication by PTGS2. (**A**) Viral replication measured in RNA copy number per ml of mock-transfected (empty vector) and pCMV-PTGS2-transfected (PTGS2) PBMC. (**B**) Protein expression of Western-blot of 20 µg lysate protein of mock-transfected (lane 1) or PTGS2-transfected PBMC (lane 2). PBMC were transiently transfected with plasmid DNA. After 2 days cells were infected with a 0.01 MOI of the HIV-1 primary isolate (HIV 89.6) for 2 hrs at 37°C. Cells were washed and supernatant was collected after 48 hr. HIV-1 RNA levels were measured using the COBAS Ampliprep/COBAS Taqman 48 system for three independent experiments. For the Western analysis, transfected Jurkat cellular homogenates (20 µg/lane) were separated by SDS-PAGE. Target proteins were identified by Western analysis using polyclonal antibody generated against the PTGS2 protein. Representative data of three independent experiments are shown. (**C**) PBMC were infected with HIV-1 primary isolate (HIV 89.6) at a MOI of 0.01. Hep-ATIII was added at concentrations of 0.09, 0.17 and 0.4 µM. After 48 hours, PTGS2 was quantified using the PTGS2-specific ELISA.

In order to assess the potential of PTGS2 to independently impact HIV-1 infection, we transiently expressed the human PTGS2 protein in human PBMC cells. We observed from transfection experiments that a 10-fold increase in PTGS2 expression could be reproducibly achieved. When PTGS2-transfected cells were incubated with HIV-1, a 50% reduction of HIV-1 replication (HIV RNA copies/ml) was observed after 48 hrs as compared to controls transfected with an empty DNA vector (**Fig. 6A and B**). We also conducted experiments to measure the kinetics of PTGS2 during viral infection at various doses of ATIII. We found that PTGS2 expression increased in a dose-dependent manner at 48 hours, and this was specific for HIV-infected cells (**Fig. 6C**).

In summary our results demonstrate (1) that PTGS2 is potently activated by ATIII in virus-infected cells, (2) that PTGS2 over-expression can significantly reduce virus production in HIV-susceptible cells *in vitro*, and (3) that PTGS2 might act as an HIV inhibitory host cell factor by direct or indirect interference with factors required for productive virus replication.

## Discussion

Serpins have a plethora of functions [7], [8], [9], [10], [11], [12], [13], [14]. It was recently demonstrated that serpins are potential inhibitors of HIV-1 replication in the blood and mucosa [16]: (1) Early during HIV infection, serpin levels in the blood increase rapidly to levels in the range of their *in vitro* inhibitory activity [32]; (2) The serpin, secretory leukocyte inhibitor (SLPI), has been described as an important HIV-1 inhibitory factor in saliva [33]; (3) serpins are highly expressed in the cervical fluids of exposed but uninfected sex workers [19], and (4) serpins are highly expressed in HIV long-term non-progressors [20], [24].

We, thus, initiated an investigation of the antiviral properties of the serpin ATIII. We demonstrated that ATIII can inhibit a variety of disparate viruses: HIV-1, HCV, HSV-1 and HSV-2. We subsequently focused our investigation on identifying gene-expression patterns that are specifically associated with ATIII treatment of HIV-infected cells in an attempt to identify host-cell factors which might act down-stream of ATIII. We decided to focus our study on HIV-1 under the assumption that the mechanism of action of ATIII might not be identical for different viruses.

To begin to understand the anti-HIV mechanism of action of ATIII, we used gene-expression arrays and protein network analysis to probe the signaling pathways activated by ATIII. This gene expression analysis indicated that the mechanism of serpin-mediated viral inhibition is multifactorial, involving a number of prominent signal transduction cascades. Furthermore, our network analysis has indicated that the proteins involved in reducing HIV replication may derive from multiple intersecting signaling pathways, although there is likely to be some level of redundancy in these regulatory and signaling cascades.

We found that ATIII induced significantly more comprehensive transcriptional changes in virus-infected cells than in uninfected cells. This apparent specificity in the mechanism of ATIII may be used to clinical advantage, as it should minimize cytotoxicity in bystander cells and thus reduce the possibility of side effects of ATIII treatment.

Among the transcripts regulated by ATIII, PTGS2 was distinguished as being most potently up-regulated by ATIII. To determine if PTGS2 activation alone might be sufficient to confer the observed anti-viral effect of ATIII, we over-expressed PTGS2 in infected cells. We found that the over-expression of PTGS2 reduced the replication of HIV in primary cells, although not to the extent observed with ATIII. The reason for this might be that we never achieved the increase of expression using the PTGS2-DNA plasmid as seen for the activation of PTGS2 expression by hep-ATIII. Furthermore, it is possible that there are additional inhibitory factors involved.

It has been previously demonstrated that ATIII, at least in part, exerts its anti-inflammatory effect by induction of prostacyclins, a family of eicosanoids acting in autocrine and paracrine pathways [2]. PTSG2 enzymatically synthesizes the eicosanoid prostaglandin H2. Another eicosanoid, prostaglandin A1 has also been shown to inhibit HSV 1 and 2 replication [34]. It is therefore possible that the observed inhibition of different viruses by ATIII is the result of downstream synthesis of eicosanoids mediated by PTGS2.

PTGS2 is known to down-regulate NF-κB mediated transcription, which is a critical element in HIV-1 replication [2]. Efficient HIV-1 gene expression depends on NF-κB to activate viral transcription. The promoter-proximal (enhancer) region of the HIV-1 long terminal repeat (LTR) contains two adjacent NF-κB binding sites (−109 to −79) that play a central role in mediating inducible HIV-1 replication. In addition to HIV-1, NF-κB is activated by several families of viruses, including HTLV-1, hepatitis B virus (HBV), hepatitis C virus (HCV), EBV and influenza virus. This activation serves to maximize virus genome production, abort the apoptosis of infected cells, and forestall immune responses to the invading pathogen.

We propose that ATIII inhibits HIV-1 replication through activation of PTGS2, which then modulates the NF-κB pathway. Our network data indicate that in addition to the NF-κB transcriptional network, the TNF transcriptional network may also be affected by PTGS2. The importance of the TNF network in the function of serpins has previously been described [31]. Our studies do not exclude other factors aside from PTGS2 being involved in ATIII-mediated virus inhibition. However, both our gene array and network analysis suggest a role for pharmacologic stimulation of PTGS2 in HIV-1 target cells as a means of interdicting the NF-κB cascade and blocking viral replication. Further investigation into the mechanism of action of PTGS2 and other proteins regulated by ATIII therapy might provide new insights into the development of anti-viral therapeutics or prophylactic agents.
